# Aneurismal subarachnoid hemorrhage in a Chilean population, with emphasis on risk factors

**DOI:** 10.1186/1756-0500-4-464

**Published:** 2011-10-28

**Authors:** Mónica Y Acuña, Lucía A Cifuentes

**Affiliations:** 1 Programa de Genética, ICBM, Facultad de Medicina, Universidad de Chile, Independencia 1027, Santiago, Stgo 8320000, Chile

## Abstract

**Background:**

Subarachnoid Hemorrhage (SAH) is caused principally by the rupture of intracranial aneurisms. Important risk factors have been described such as age, sex, hypertension (HT) and season of the year, among others. The objective is to investigate the demographic characteristics and possible risk factors in a population of Chilean patients.

**Methods:**

This retrospective study was based on the analysis of 244 clinical records of patients diagnosed with aneurismal SAH who were discharged from the Instituto de Neurocirugía ASENJO in Santiago, Chile.

**Results:**

The mean age of patients was 49.85 years and the male:female ratio was 1:2.7. The signs and symptoms were not different between sexes; cephalea (85.7%) was predominant, followed by loss of consciousness, vomiting/nausea and meningeal signs. Risk factors included sex, age and HT. Concordant with other reports, the incidence of SAH was greatest in spring.

**Conclusions:**

The demographic characteristics and risk factors observed in patients with aneurismal SAH treated in ASENJO were comparable to those of other populations. We were not able to conclude that tobacco and alcohol consumption were risk factors for this population.

## Background

Subarachnoid hemorrhage (SAH) is a clinical entity characterized by the presence of blood in the subarachnoid space. The principal cause of spontaneous SAH is the rupture of intracranial aneurisms (75-80% of cases), followed by idiopathies (14-22%) and arterio-venous malformations (4-5%) [1,2].

There is currently high precision in the diagnosis of SAH, which has allowed it to be described at the population level; the incidence varies between 1.4 and 24.8 cases per year per 100,000 individuals [3]. Kuwait [4] has the lowest reported incidence of SAH and Japan [5] the highest. Most countries in Europe have rates between 2.8 and 11.8; however, Finland has 19.7 and Denmark 16.6 [3]. The PISCIS study performed in Iquique, Chile, found an incidence of 3.8 per 100,000 inhabitants per year [6].

Rooij et al. [3] analyzed the incidence of SAH aneurisms in 20 of 51 studies in different populations. Using the incidence found in the age range 45-55 years as a reference, they indicated that the relative incidence increased from 0.10 for persons < 25 years to 1.61 for those ≥ 85 years old. Also these authors found a mean rate of 9.2 for men and 11.5 (1.24 times greater) for women; in the age range of 25-45 the incidence was significantly greater in men, while the converse was true in the range 55-85.

SAH is found in 4-7% of all cerebral vascular accidents; it has a high mortality and has one of the worst prognoses [1,2]. The etiology of SAH is poorly understood. Smoking and arterial hypertension have been reported as the most important risk factors; cholesterolemia, alcohol consumption and genetic makeup have also been implicated. There is still controversy about these risk factors [7-10]. Another risk factor which has been indicated for SAH is the time of year and the time of day; atmospheric pressure has been suggested as a determinant factor in aneurismal SAH, while the literature indicates that this rupture occurs more frequently in spring [11-14].

The objectives of this report are: a) to describe the frequency and behavior of aneurismal SAH by age, sex and season in which the ruptures occur, and to investigate whether socioeconomic stratum has any relation to aneurismal SAH [15], in a sample of the records of patients who were treated in the Instituto de Neurocirugía ASENJO of the Servicio de Salud Metropolitano Oriente, Santiago, from January 1995 to June 1997; b) to compare the frequency of occurrence of potential risk factors (HT, alcohol consumption, smoking and obesity) to the frequencies of a control population [16]; and c) to describe the most frequent symptoms of patients with aneurismal SAH in the sample and to determine if there are differences between sexes.

## Methods

This study was performed with the clinical records of patients who were diagnosed with aneurismal SAH and were treated in the Instituto de Neurocirugía ASENJO of the Servicio de Salud Metropolitano Oriente, Santiago, from January 1995 to June 1997. This is the only public hospital in Chile which treats SAH; patients are referred here from all over the country. ASENJO treats 80% of the cases of aneurismal SAH in Chile; the remaining 20% are treated in private clinics. We selected all ASENJO patients, without regard to the etiology of the aneurism, in order to avoid the loss of information due to misclassification or omission of the cause of bleeding. Revision of clinical records was made under institutionally approved internal hospital protocols with oral informed consent. The study was approved by the Ethics Committee for Human Research of the Facultad de Medicina of the Universidad de Chile.

From a total of 420 records searched for in the archives of ASENJO, 34 were not found and 142 were not due to aneurismal SAH; 244 were patients who did have aneurismal SAH, confirmed by angiogram or surgery. Of this last group, 178 (73%) were women and 66 (27%) were men.

The following information was extracted from the clinical records: sex, age, type of medical insurance, season of the year of the aneurism, symptoms and signs, time between admission and discharge from the hospital, risk factors, Glasgow, tests performed, Fisher classification, number and location of aneurisms, treatment, presence of vasospasm pre- and post-operation and condition at discharge. The type of medical insurance was used as a socioeconomic indicator, using the six classes which are used by the National Health Fund (ex-FONASA) according to the income level of the patient.

The HT risk factor was classified using the three categories which the doctor recorded in the anamnesis at admission: a) normal, those with normal arterial pressure (AP); b) hypertensive without treatment, including those patients with high AP who knew of their condition but had not received treatment and those who did not know they were hypertensive; and c) hypertensive patients in treatment.

Consumption of alcohol and obesity were recorded if indicated in the clinical record. Tobacco use was recorded as non-smokers and smokers (daily cigarette smoking without regard to quantity). Finally, the season of the year in which aneurism rupture occurred was classified as summer (21 December to 20 March), autumn (21 March to 20 June), winter (21 June to 20 September) or spring (21 September to 20 December). This was done for the years 1995 and 1996; we excluded the 7 months of 1997.

We used a published study of risk factors for chronic illnesses for the general population of the Santiago area as a control (reference) population [16]. This study included the prevalence and behavior of HT, smoking, obesity and alcohol consumption by age, sex and socioeconomic stratum in a sample from 1986-1987.

We estimated the statistical significance of the risk factors by calculating the odds ratios (OR) and their confidence intervals. If these intervals do not include 1, they are significant [17]. We also used the Z test for proportions and Chi square, in accordance with the data [18,19].

## Results

The mean age of patients in the sample was 49.85 years; 47.5 for males and 52.0 for females. 18.8% of the patients were of less than 40 years; the minimum was 2 years and the maximum 80. The majority of the patients were from the low socioeconomic stratum; 69.1% belonged to the three lowest groups of the National Health Service, and only 19.1% had private health care.

The ratio of males to females was 1:2.7; there were 178 females (73%) and 66 males (27%) among the 244 patients (Table 1). Aneurismal SAH was very infrequent before age 20 (2.46%); it increased in frequency with increase in age, reached a maximum between ages 40-60 (56.97%) and decreased with greater age. At less than age 20, the frequency of aneurismal SAH was the same in both sexes (50%). After age 20 there was an increase in female cases; in the 20-30 age range the percentage was almost double that of the males, and between ages 60-70 it was six times greater in women.

**Table 1:** Ration Woman/Man and Distribution of Patients with Aneurismal SAH According to Age, Attended in Asenjo.

EDAD	♂		♀			♀♂	
	
	N	%	N	%	♀/♂	N	%
**< 20**	3	50.00	3	50.00	1.00	6	2.46
**20.1 - 30**	4	33.33	8	66.67	2.00	12	4.92
**30.1 - 40**	9	32.14	19	67.86	2.10	28	11.48
**40.1 - 50**	20	28.17	51	71.83	2.55	71	29.10
**50.1 - 60**	20	29.41	48	70.59	2.40	68	27.87
**60.1 - 70**	6	13.64	38	86.36	6.33	44	18.03
**70.1 - 80**	4	26.67	11	73.33	2.75	15	6.15
**TOTAL**	**66**	**27**	**178**	**73**	**2.70**	**244**	**100**

Santiago, Chile, from January 1995 to July 1997.

Table 2 summarizes the frequencies of symptoms and initial signs by sex in patients admitted for aneurismal SAH in ASENJO. The frequencies were calculated based on the total number of symptoms and signs present in each sex. These were cephalea, vomiting/nausea, loss of consciousness, meningeal signs and hemiparesis. Their frequencies were similar in males and females. The most frequent symptom was cephalea (85.65%), followed by loss of consciousness (50%), vomiting/nausea (47.13%) and meningeal signs (28.68%). Other symptoms and signs were found at low frequencies.

**Table 2:** Frequency of Symptoms and Signs Shown by Patients Admited for Aneurismal SAH by Sex, Asenjo, Santiago (1995 - 1997)

SYMPTOMS AND SIGNS	♂		♀		♀♂	
	
	N	%	N	%	N	%
**Cephalea**	56	36.13	153	36.86	209	85.65
**Vomiting/nausea**	29	18.71	86	20.72	115	47.13
**Loss of consciousness**	34	21.94	88	21.20	122	50.00
**Meníngeal signs**	21	13.55	49	11.81	70	28.68
**Hemiparesia**	4	2.58	12	2.89	16	6.55
**Language difficulty **	5	3.23	1	0.24	6	2.45
**Convulsions**	2	1.29	10	2.40	12	4.91
**Cochleovestibular alterations**	1	0.65	6	1.45	7	2.86
**Oculomotor alteration**	3	1.93	6	1.45	9	3.68
**Relaxation of sphincter**	0	0	1	0.24	1	0.40
**HT crisis**	0	0	3	0.72	3	1.23
**TOTAL**	**155**	**100**	**415**	**100**	**570**	**100**

Table 3 shows the observed and expected distribution of aneurism rupture by sex and by season. There was a peak of ruptures in the spring (35.4%) and fewer ruptures in winter (18.2%) and summer (20.1%). These differences are significant (χ^2^_calc. _= 15.09; 3 g.l; p_v _= 0.0017). The percentage of ruptures was greatest in spring for both men and women.

**Table 3:** Observed (O) and Expected (E) Frequency of Rupture of Aneurisms by Sex and Season of the Year in Patients Attended for Aneurismal SAH, Asenjo from January 1995 to December 1996.

	♂				♀				♀♂			
	
SEASON OF THE YEAR	№	% O	№ E	χ^2^	№	% O	№ E	χ^2^	№	% O	№ E	χ^2^
**AUTUMN**	11	19.30	14.25	0.74	44	28.95	38	0.95	55	26.3	52.25	0.14
**WINTER**	9	15.79	14.25	1.93	29	19.08	38	2.13	38	18.2	52.25	3.86
**SPRING**	23	40.35	14.25	5.37	51	33.55	38	4.45	74	35.4	52.25	9.05
**SUMMER**	14	24.56	14.25	0.00	28	18.42	38	2.63	42	20.1	52.25	2.01

**TOTAL**	**57**	**100**	**57**	**8.05**	**152**	100	**152**	**10.16**	**209**	**100**	**209**	**15.06**

**p value**				0.045				0.017				0.0017

Table 4 gives the distribution of the aneurismal ruptures classified by season, age and sex. There were differences between sexes in the presentation of aneurismal SAH among seasons. Men had the highest frequency in spring (40.35%) followed by summer (24.56%), while women had the highest frequency in spring (33.55%) and autumn (28.95%), and the lowest frequency in summer (18.42%).

**Table 4:** Seasonal Distribution of Ruptures of Aneurisms in Patients Attended for HSA in Asenjo by Age and Sex from January 1995 to December 1996

	MEN									
	
	AUTUMN		WINTER		SPRING		SUMMER		TOTAL	
AGE	N	%	N	%	N	%	N	%	N	%
< 20	0	0.00	1	50.00	0	0.00	1	50.00	2	50.00
20.1 - 30	1	25.00	0	0.00	2	50.00	1	25.00	4	33.33
30.1 - 40	2	25.00	1	12.50	2	25.00	3	37.50	8	38.10
40.1 - 50	3	17.65	3	17.65	7	41.18	4	23.53	17	30.91
50.1 - 60	2	11.76	3	17.65	10	58.82	2	11.76	17	29.82
60.1 - 70	2	33.33	1	16.67	2	33.33	1	16.67	6	14.29
70.1 - 80	1	33.33	0	0.00	0	0.00	2	66.67	3	16.67
TOTAL	11	19.30	9	15.79	23	40.35	14	24.56	57	27.27
										
	χ^2^_cal _= 28.61; P_v _= 0.053									
										
	**WOMEN**									
	
	**AUTUMN**		**WINTER**		**SPRING**		**SUMMER**		**TOTAL**	
**AGE**	**n**	**%**	**n**	**%**	**n**	**%**	**n**	**%**	**N**	**%**
**< 20**	2	100.00	0	0.00	0	0.00	0	0.00	2	50.00
**20.1 - 30**	5	62.50	0	0.00	2	25.00	1	12.50	8	66.67
**30.1 - 40**	5	38.46	1	7.69	7	53.85	0	0.00	13	61.90
**40.1 - 50**	12	31.58	11	28.95	14	36.84	1	2.63	38	69.09
**50.1 - 60**	8	20.00	9	22.50	13	32.50	10	25.00	40	70.18
**60.1 - 70**	9	25.00	5	13.89	11	30.56	11	30.56	36	85.71
**70.1 - 80**	3	20.00	3	20.00	4	26.67	5	33.33	15	83.33
**TOTAL**	**44**	**28.95**	**29**	**19.08**	**51**	**33.55**	**28**	**18.42**	**152**	**72.73**
										
	**χ^2^_cal _= 30.87; P_v _= 0.03**									

										
**TOTAL (♂♀)**	**55**	**26.31**	**38**	**18.18**	**74**	**35.4**	**42**	**20.09**	**209**	**100**
										
	**χ^2^_cal _= 15.1; P_v _= 0.0017**									

Men between 40 and 50 years of age had the highest frequency of aneurismal SAH in spring (41.18%) and summer (23.53%), and the lowest frequency in autumn and winter (both 17.65%). Men of age 50-60 also had the highest frequency of ruptures in spring (58.82%), and a much lower frequency in winter (17.65%), autumn and summer (both 11.76%). Men of age 60-70 had 33.33% in spring and autumn, and 16.67% in winter and summer. Above 70 years of age, the most important season for aneurismal SAH was summer (66.67%) followed by autumn (33.33%). Women younger than 30 years had more cases in autumn, but at older ages there were more aneurismal ruptures in spring. The seasonal frequency of aneurismal SAH was not significantly different among age classes for men (χ^2 ^= 13.37; P_V _= 0.8191), but was significant for women (χ^2 ^= 30.87; P_V _= 0.03). Combining the results for spring and autumn and for summer and winter, the difference was significant for women but not for men (χ^2 ^= 12.78; P_V _= 0.03).

Table 5 compares the frequency of the different risk factors between patients with aneurismal SAH and the control group R.M. Of the 66 male patients of ASENJO, 26 had hypertension (39.39%) while 50.56% of the women (90 out of 178) had hypertension; this difference is at the limit of statistical significance (p = 0.057). It is worth noting that above age 65 there was an increase of the observed frequency of HT in males compared to females. By contrast, the control group had similar total frequencies of HT for the two sexes; although under age 54 the frequency of HT was greater in men, while the reverse was true for those 55 and older. Among men, HT was present in all age ranges of the R.M. sample, while in ASENJO patients it was only present above age 35, with frequencies significantly greater than the control group (Z_calc _= 5; p = 2.85 × 10 ^-7^). By contrast, the frequencies of HT observed among women in the ASENJO sample were significantly greater than those of the control sample beginning at age 25 (Z_calc _= 10; p = 0). In the ASENJO sample HT appeared in women at an earlier age than in men. The OR and confidence intervals for SAH in men and women with hypertension were 6.9 [3.67<OR<12.64] and 10.26 [3.67<OR<12.64], respectively.

**Table 5:** Comparison of the Frequencies of Risk Factors by Age Group in Patients Who are Carriers of Aneurismal SAH in Asenjo and a Control Sample of the Metropolitan Region (RM)

		MEN ASENJO			**MEN R.M**.			WOMEN ASENJO			**WOMEN R.M**.			
		
	AGE	n	%	№ studied	n	%	№ studied	n	%	№ studied	n	%	№ studied	
	
	< 24	0	0.00	6	1	0.70	142	0	0.00	4	0	0.00	171	
	25 - 34	0	0.00	2	2	1.98	101	4	28.57	14	1	0.56	179	
	35 - 44	3	23.08	13	6	7.14	84	10	38.46	26	3	2.29	131	
	45 - 54	13	44.83	29	10	14.71	68	28	51.85	54	14	12.84	109	
	55 - 64	4	44.44	9	11	21.57	51	30	55.56	54	18	23.08	78	
	≥ 65	6	85.71	7	11	37.93	29	18	69.23	26	30	50.00	60	
HT	Total	26	39.39	66	41	8.63	475	90	50.6	178	66	9.07	728	
														
		**MEN ASENJO**			**MEN R.M**.			**WOMEN ASENJO**			**WOMEN R.M**.			
		
	**AGE**	**n**	**%**	**№ studied**	**n**	**%**	**№ studied**	**n**	**%**	**№ studied**	**n**	**%**	**№ studied**	
	
	**< 24**	1	16.67	6	65	45.77	142	0	0.00	4	31	18.13	171	
	**25 - 34**	1	50.00	2	62	61.39	101	0	0.00	14	45	25.14	179	
	**35 - 44**	1	7.69	13	52	61.90	84	2	7.69	26	29	22.14	131	
	**45 - 54**	3	10.34	29	46	67.65	68	4	7.41	54	19	17.43	109	
	**55 - 64**	3	33.33	9	27	52.94	51	1	1.85	54	15	19.23	78	
	**≥ 65**	1	14.29	7	15	51.72	29	0	0.00	26	5	8.33	60	
**ALCOHOL**	**Total**	**10**	**15.15**	**66**	**267**	**56.21**	**475**	**7**	**3.93**	**178**	**144**	**19.78**	**728**	

														
		**MEN ASENJO**			**MEN R.M**.			**WOMEN ASENJO**			**WOMEN R.M**.			
		
	**AGE**	**n**	**%**	**№ studied**	**n**	**%**	**№ studied**	**n**	**%**	**№ studied**	**n**	**%**	**№ studied**	
	
	**< 24**	3	50.00	6	73	51.41	142	0	0.00	4	85	49.71	171	
	**25 - 34**	0	0.00	2	73	72.28	101	1	7.14	14	102	56.98	179	
	**35 - 44**	2	15.38	13	42	50.00	84	6	23.08	26	68	51.91	131	
	**45 - 54**	8	27.59	29	36	52.94	68	8	14.81	54	36	33.03	109	
	**55 - 64**	4	44.44	9	13	25.49	51	9	16.67	54	22	28.21	78	
	**≥ 65**	0	0.00	7	4	13.79	29	1	3.85	26	3	5.00	60	
**SMOKER**	**Total**	**17**	**25.76**	**66**	**241**	**50.73**	**475**	**25**	**14**	**178**	**316**	**43.41**	**728**	

														
		**MEN ASENJO**			**MEN R.M**.			**WOMEN ASENJO**			**WOMEN R.M**.			
		
	**AGE**	**n**	**%**	**№ studied**	**n**	**%**	**№ studied**	**n**	**%**	**№ studied**	**n**	**%**	**№ studied**	
	
	**< 24**	0	0.00	6	1	0.70	142	0	0.00	4	13	7.60	171	
	**25 - 34**	0	0.00	2	10	9.90	101	0	0.00	14	31	17.32	179	
	**35 - 44**	0	0.00	13	14	16.67	84	1	3.85	26	32	24.43	131	
	**45 - 54**	0	0.00	29	14	20.59	68	4	7.41	54	38	34.86	109	
	**55 - 64**	2	22.22	9	20	39.22	51	3	5.56	54	28	35.90	78	
	**≥ 65**	0	0.00	7	3	10.34	29	2	7.69	26	23	38.33	60	
**OBESE**	**TOTAL**	**2**	**3.03**	**66**	**62**	**13.05**	**475**	**10**	**5.62**	**178**	**165**	**22.66**	**728**	

There was greater alcohol consumption in men at all ages in both groups. Alcohol consumption was significantly greater in the control group than among ASENJO patients in both sexes and in all age groups. This factor was present in men in ASENJO sample at all ages, while in women it was found only above age 35 (OR_alcohol♂ _= 0.14; [0.06<OR<0.29], OR_alcohol♀ _= 0.17; [0.07<OR<0.37]). Tobacco use was greater among men in both samples; however, tobacco use was significantly lower in ASENJO patients (25.76% in men and 14.04% in women) than in the control sample (50.73% in men and 43.41% in women). The odds ratios were OR_tobacco♂ _= 0.34; [0.18<OR<0.62], OR_tobacco♀ _= 0.21; [0.03<OR<0.90]. Finally, there were obese persons of both sexes in all age classes in the control group, whose percentages were significantly greater than in the ASENJO group (OR_obesity♂ _= 0.21; [0.03<OR<0.9]; OR_obesity♀ _= 0.21; [0.1<OR<0.43]). All the odds ratios given above were statistically significant.

## Discussion

In Chile, a cerebrovascular attack (CVA) is the second most common cause of death (46.3 per 100,000 inhabitants); it is one of the illnesses which produce the greatest incapacity in Chile and in the world [1,5,20]. Between 3 and 5% of CVA are due to aneurismal SAH. Although this is a small percentage, its social impact is important because a significant proportion occurs in persons less than age 40 (we found 18.85%), many of whom die or lose years of productive life [1,2].

It should be emphasized that this is a retrospective study which analyzed data from the years 1995 to 1997 of the Instituto de Neurocirugía ASENJO. A great advantage of performing studies in base hospitals such as this is that of obtaining a relatively large number of patients with SAH. However, patients hospitalized with SAH may not be a representative sample of SAH sufferers, since some patients die before reaching the hospital [1,11].

### Social stratum and aneurismal SAH

We did not find an association between socioeconomic statrum and aneurismal SAH; however, the great majority of the patients in our sample were from the low socioeconomic class. An unpublished undergraduate thesis [21] which studied aneurismal SAH in the Clinica Las Condes, a private hospital serving principally the high socioeconomic class found frequencies similar to those of this study (data not shown); thus an association of social stratum and aneurismal SAH is unlikely.

### Age and aneurismal SAH

The rupture of aneurisms may occur at any age, but is more frequent after age 45 [2,3]. In our sample, 75% of the cases were between age 40 and 70; the peak was in the 40-50 range and the frequency declined above age 60 years. The OR calculated by age groups, using the age distribution of the control RM sample [16], showed that younger age groups were relatively protected OR_15-24 years _= 0.12 [0.06<OR<0.24] and OR_25-34 years _= 0.15 [0.08<OR<0.28], while the older groups were at high risk (OR_45-54 years _= 2.99 [2.17<OR<4.12], OR_55-64 years _= 2.9 [2.03<OR<4.13] and OR_65-more years _= 1.96 [1.25<OR<3.06]). The OR_35-45 _= 0.87 [0.59<OR<1.29] was not significant. Our results are in agreement with those of previous studies [1-5,7-11,22,23].

### Sex and aneurismal SAH

In the majority of epidemiological studies the female/male ratio is greater than one, and increases with increasing age of women [1,2,22-28]. There was a significant preponderance of women in our sample (2.7:1), and the female/male ratio increased with increasing age of the women. As in other studies [2,3,22-28], being a woman is a risk factor OR = 1.76; [1.28<OR<2.42].

### HT and aneurismal SAH

HT is a well known risk factor in the formation, development and rupture of intracranial aneurisms [2,7,8,10,24-26,28]. HT was a relevant risk factor for the studied population, and was more important for women than for men. Of the population served by the Instituto de Neurocirugía ASENJO, 47.5% of the cases of aneurismal SAH could be attributed to a systolic and/or diastolic blood pressure above 160 mmHg and/or 95 mmHg, respectively, as a principal factor. This suggests that a substantial portion of aneurismal SAH cases could be prevented by reducing arterial pressure, especially considering that an important number of the patients are younger people.

### Obesity and aneurismal SAH

A number of studies have found that the body mass index (BMI = weight/height^2^) is inversely associated with the risk of aneurismal SAH [7,10,11]. Although we only classified patients as obese or not obese, we found the same association; thus excess weight appears to be a protective factor for aneurismal SAH for the population served by the Instituto de Neurocirugía ASENJO.

### Tobacco and alcohol use and aneurismal SAH

The use of tobacco and alcohol consumption have both been recognized as risk factors for the formation and rupture of aneurisms [7,10,11,25,27,28]. Our results show the contrary; however, the data obtained from clinical records are not trustworthy, since the use of both drugs may be purposefully hidden by the patients or because it was impossible to obtain this information directly from them.

### Seasonal variation in aneurismal SAH

As in the cases of aneurisms of the abdominal aorta and acute myocardial infarctions, a seasonal variation has been described in the incidence of ruptures of cerebral aneurisms [29]. We found, as have studies in a number of countries, that more ruptures occur in spring [11-14,29]. It is not yet clear what the influence of the season on the rupture of intracranial aneurisms may be, however changes in barometric pressure have been suggested as an important factor [11-14,29].

## Conclusions

The demographic characteristics and the risk factors observed in patients with aneurismal SAH attended by the Instituto de Neurocirugía ASENJO are compatible with those present in other populations, although we could not conclude that alcohol and tobacco consumption are risk factors in the Chilean population.

## Competing interests

The authors declare that they have no competing interests.

## Authors' contributions

MA designed the research, carried out the analyses and wrote the manuscript. LC assisted in revising the manuscript. Both authors read the paper and approved the final version.
